# Noncontact recognition of fluorescently labeled objects in deep tissue via a novel optical light beam arrangement

**DOI:** 10.1371/journal.pone.0208236

**Published:** 2018-12-19

**Authors:** Andreas Hien, Marc Pretze, Frank Braun, Edgar Schäfer, Tim Kümmel, Mareike Roscher, Daniel Schock-Kusch, Jens Waldeck, Bernhard Müller, Carmen Wängler, Matthias Rädle, Björn Wängler

**Affiliations:** 1 Institute of Process Control and Innovative Energy Conversion, Mannheim University of Applied Sciences, Mannheim, Germany; 2 Molecular Imaging and Radiochemistry, Department of Clinical Radiology and Nuclear Medicine, Medical Faculty Mannheim of Heidelberg University, Mannheim, Germany; 3 Bruker BioSpin MRI GmbH, Ettlingen, Germany; 4 ProxiVision GmbH, Bensheim, Germany; 5 Biomedical Chemistry, Department of Clinical Radiology and Nuclear Medicine, Medical Faculty Mannheim of Heidelberg University, Mannheim, Germany; University of Campinas, BRAZIL

## Abstract

To date, few optical imaging systems are available in clinical practice to perform noninvasive measurements transcutaneously. Instead, functional imaging is performed using ionizing radiation or intense magnetic fields in most cases. The applicability of fluorescence imaging (e.g., for the detection of fluorescently labeled objects, such as tumors) is limited due to the restricted tissue penetration of light and the required long exposure time. Thus, the development of highly sensitive and easily manageable instruments is necessary to broaden the utility of optical imaging. To advance these developments, an improved fluorescence imaging system was designed in this study that operates on the principle of noncontact laser-induced fluorescence and enables the detection of fluorescence from deeper tissue layers as well as real-time imaging. The high performance of the developed optical laser scanner results from the combination of specific point illumination, an intensified charge-coupled device (ICCD) detector with a novel light trap, and a filtering strategy. The suitability of the laser scanner was demonstrated in two representative applications and an in vivo evaluation. In addition, a comparison with a planar imaging system was performed. The results show that the exposure time with the developed laser scanner can be reduced to a few milliseconds during measurements with a penetration depth of up to 32 mm. Due to these short exposure times, real-time fluorescence imaging can be easily achieved. The ability to measure fluorescence from deep tissue layers enables clinically relevant applications, such as the detection of fluorescently labeled malignant tumors.

## Introduction

The International Cancer Research Agency has estimated that more than 8.2 million people died of cancer in 2012 [1]. Cancer is therefore the second leading cause of death [2]. Bray et al. predicted from demographic trends based on United Nations estimations that 22.2 million new cancer cases will occur by 2030 [3]. Early detection of malignant tumors is important as it improves the chance of recovery from the disease [4]. Tissue changes due to cancer can be identified noninvasively by imaging techniques such as computed tomography (CT), positron emission tomography (PET), magnetic resonance imaging (MRI) and optical imaging. This work focuses on optical imaging, which is important for detection of, e.g., breast cancer, melanoma and near-surface lesions and for optical guidance in tumor therapy. The advantage of optical imaging is that it does not use ionizing radiation or expensive equipment and does not entail high operating and maintenance costs, unlike CT, PET or MRI [5–7]. Optical imaging provides a highly sensitive and cost-effective imaging technique for use with tissues and diseases and is therefore worth exploring further [4,7–11]. In particular, fluorescence imaging is a highly sensitive and functional imaging process that enables high contrast enhancement between tumors and the surrounding healthy tissue [8–12]. The principle is based on laser-induced fluorescence [13–16]. However, when using optical methods, the depth of light penetration into the skin or tissue is limited [8,17,18]. At present, only near-surface lesions can be detected by such methods unless they are imaged intraoperatively [19]. In fluorescence imaging, a common problem is that light from the secondary light path is multiply scattered due to the tissue properties, and therefore, the objects cannot be sharply imaged. This reduces the resolution of the imaging system. Detected fluorescent objects are difficult to resolve in detail. Therefore, few applications in clinical imaging currently rely on fluorescence measurement techniques [20–24]. Consequently, whole-body optical imaging to date is only suitable in preclinical settings [25–27]. In currently available optical imaging systems, high fluorescent dye concentrations and/or a high laser power combined with a sensitive low-noise charge-coupled device (CCD) or complementary metal-oxide-semiconductor (CMOS) detectors are required for the imaging of deep tissue layers [10,25,28]. However, the required high excitation intensity can cause tissue overheating, leading to serious damage [29]. Thus, light in the infrared range is preferable because it is less scattered and absorbed by tissue than light in the visible spectrum, while simultaneously providing sufficient emission signals. In particular, three areas called optical windows, which are located in areas of 850 nm, 1300 nm and 1550 nm, provide favorable conditions for deep penetration into tissue [1, 2]. Nevertheless, the penetration depth of continuous-wave (CW) optical imaging systems is often less than 1.5 cm [30–34]. An additional disadvantage of the present noncontact CW in vivo optical imaging systems is that their low signal intensities require the use of long integration times to produce acceptable images with sufficient signal-to-noise ratios (SNRs) [33].

Various other microscopic and macroscopic methods involve the use of time-domain techniques, such as time-domain photon migration (TDPM), frequency-domain photon migration (FDPM) or multiphoton microscopy [18,34–39,40]. The fields of application of these techniques can be found, for example, in the 3D representation of tumors or the representation of high-resolution depth-localized tissue images [41]. These techniques are based on a knowledge of the excited electronic and vibrational states of the fluorescent molecules. In these methods, the lasers must be pulsed at frequencies on the order of pico- or femtoseconds, and the elastically scattered light can be separated from the fluorescent emission [38,42]. Using these techniques, a higher penetration depth can be achieved than that obtained using CW methods. However, numerous pulses are required due to the necessary exposure time of approximately 5–30 s [18,37,38]. Furthermore, the camera electronic components are disturbed not only by thermal noise but also by readout noise because many values must be measured; this causes additional uncertainty that impedes the acquisition of clear signals. Neither normal CCD cameras nor continuous line lasers can be used in TDPM and FDPM methods [37,39]. Thus, laser modulation techniques based on pulsed lasers and fast triggered detectors are mandatory. However, these require substantial engineering efforts to be applicable for TDPM and FDPM [35–39]. To date, no simple, cost-effective combination of lasers and synchronization detectors comparable to CW systems is available on the market [7].

In contrast to TDPM and FDPM techniques, the design developed in this work does not require the electronics needed to pulse the laser and the fast gating function of the image intensifier. In addition, the electronic components necessary for the synchronization of the laser and image intensifier in other methods are not necessary with the laser scanner used in this system. This study presents an approach that utilizes the simplest technology in the field of fluorescence research and investigates the combination of a noncontact CW fluorescence imaging system with a novel optical light path structure in a detection system that significantly increases the SNR and detection frequency. An optical structure with an optimized optical arrangement was thus developed and evaluated.

## Materials and methods

**Fig 1:**
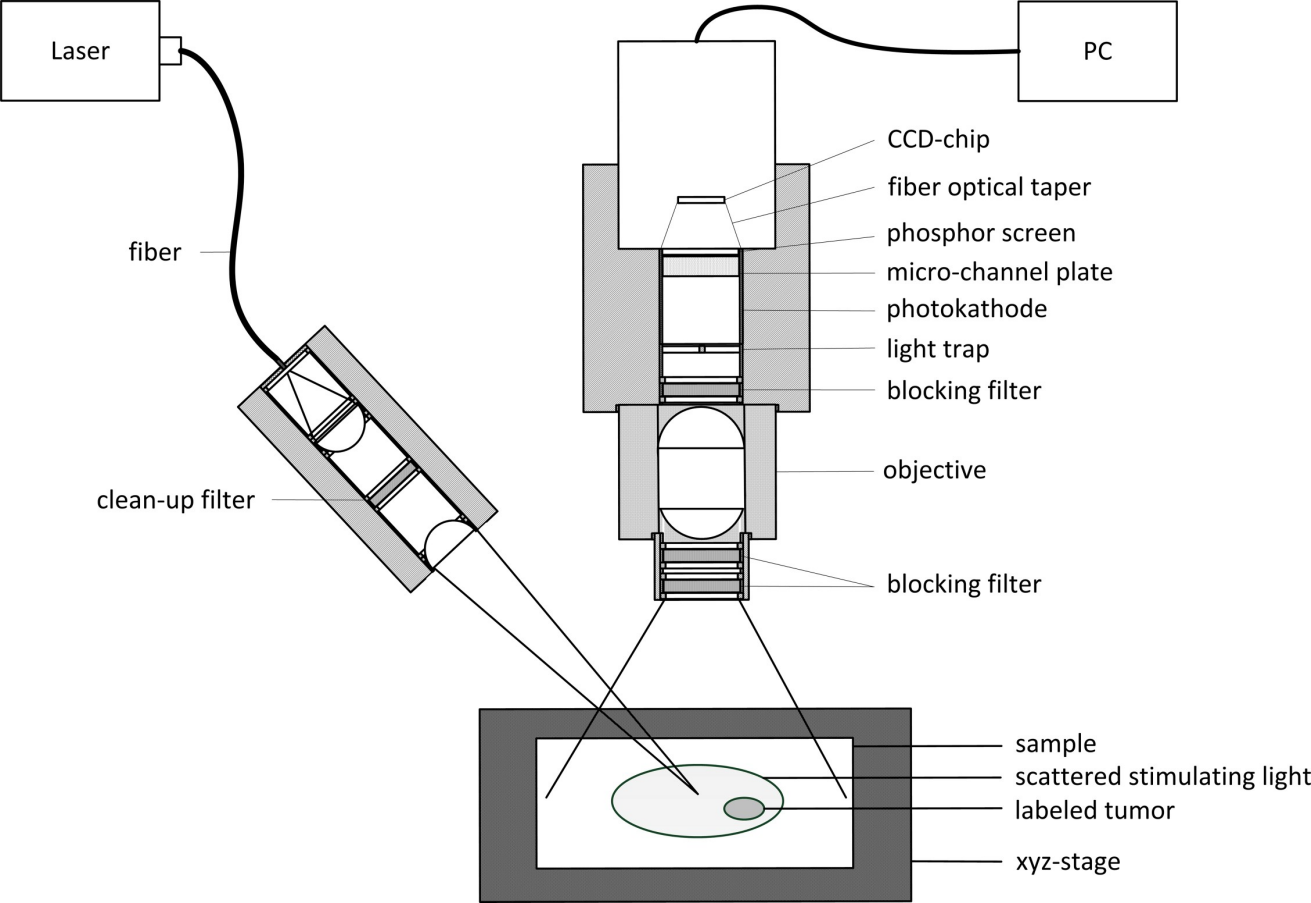
Schematic diagram of the developed fluorescence imaging system.

The fluorescence imaging configuration shown in Fig 1 was employed in this study. For the fluorescence imaging system, a stabilized single-mode laser diode (RLT785-100MGS, Roithner Laser Technik GmbH, Austria) with a CW output of 785 nm and 100 mW was chosen for fluorescence excitation. The fiber-coupled light was collimated using a plano-convex lens (LA1540-B, Thorlabs GmbH, Germany) and was then passed through a 785-nm laser cleaning filter (68–947, Edmund Optics GmbH, Germany). The narrowband filter improves the emission wavelength by blocking the side-by-side emissions of the laser diode. Subsequently, a plano-convex lens (LA1464-B, Thorlabs GmbH, Germany) was utilized to focus the excitation laser light with a spot size of 1 mm and an optical output power of approximately 17 mW directly onto the sample. A black charcoal-based coating was applied to the imager housing walls to minimize stray light and to suppress direct reflections from the samples. The primary components of the detection unit were a 12-bit CCD camera (Manta G-145, Allied Vision Technologies GmbH, Germany) and an image intensifier with a resolution of 30 μm (BV-1882-NZ, ProxiVision GmbH, Germany). The photocathode of the image intensifier was coupled to an f/1.65, 35-mm fixed-focal-length lens (35 mm C Series, Edmund Optics GmbH, Germany) to focus the detected images from the samples onto the photocathode. Image amplification was achieved using a microchannel plate. The images were projected onto a phosphor screen, and the output from the screen was coupled to the CCD sensor using a fiber optical taper (ProxiKit, ProxiVision GmbH, Germany). The image sensor of the CCD camera was used to perform vertical and horizontal binning, thereby enabling a high SNR and improving signal processing speed. To extract the desired weak fluorescence signals on the detection side, a solid blocking filter stack comprising three 810 nm long-pass filters was used (F76-787, Semrock Inc., USA). The arrangement of this filter combination, as illustrated in Fig 1, helped to minimize the detection of reflections, background light and other fluorescent substances that naturally occur in the body, such as porphyrins. An artificial iris was used to hide the surface of the elastic remission spot of the laser on the labeled sample. For this purpose, a miniaturized but efficient light trap was developed and integrated directly into the light path near the center in front of the focal plane of the amplifier. This light trap prevents overexposure of the image intensifier. For the experiments with the tumor-bearing mice, the diameter of the light trap was about 3 mm. For the pilot measurements, a larger diameter of the light trap was chosen to test the functionality of the system. This is clearly shown by S3 File in the supporting material in which the light path structure of the detection path is shown. Thereby, it was possible to focus the excitation laser light directly on the sample, as shown in Fig 1. The light trap was constructed of a polymer material with a mat surface and a high absorption coefficient to avoid light reflections.

### Detection of fluorescence from deep tissue layers

Rice grains were used as tumor phantoms in experiments involving the detection of fluorescence originating from deep tissue layers because their autofluorescence is negligible. The phantoms had a size of 7 × 2 mm and were stained [26] by immersion in an indocyanine green (ICG) solution (PULSION Medical Systems SE, Germany) [absorbance: 785 nm/emission: 830 nm] with a concentration of 3.125 μg ICG/1 mL H_2_O for 24 h in a dark and cool environment. Because the absorption coefficients of hemoglobin, lipids, and water are low at approximately 700–900 nm [9,43,44], this optical window is clearly advantageous for in vivo imaging [35,36]. To determine the maximum penetration depth, the phantoms were implanted into porcine muscle at defined intervals. The corresponding reduced scattering coefficient at 785 nm was 7.92 cm^-1^. The reduced scattering coefficient at 500 nm was 7.18 cm^-1^. The reduced scattering coefficients of muscle tissue published by Jacques et al. are between 9.8 cm^-1^ and 13.0 cm^-1^ at 500 nm [42, 45]. The excitation optic was focused on the surface of each sample at an angle of 35° relative to the detection optic, and the fluorescence was detected at a distance of 210 mm from the sample. The exposure time was set to 100 ms, binning was set to 2 × 2 pixels, and the gain of the CCD camera was set to zero. The CMOS camera (Orca Flash 4.0 Hamamatsu, Japan) used for the reference measurements was set to the same parameters as those used by the developed laser scanner.

### Fast fluorescence imaging

Experiment 2.1 fundamentally investigated whether the detection system is suitable for the detection of fluorophores in deeper tissue layers. Experiment 2.2 investigated how rapidly fluorophores can be detected in deeper tissue layers. To investigate fast fluorescence imaging at a frequency of 20 Hz, the fluorescence of tumor phantoms in animal tissue was measured using a 50-ms exposure time at a penetration depth of up to 32 mm, corresponding to real-time imaging conditions. The phantoms were prepared with ICG as described in the previous paragraph. The mechanical properties and the phantom size were also the same as those described in the previous penetration depth experiment. The camera settings differed only with respect to the exposure time. The field of view was approximately 30 mm x 20 mm. For comparison with planar imaging systems, which usually detect a large field of view, a surface scan of an sample was performed to illustrate the benefits and scope of the design developed in this project. Due to the short exposure times of the individual images with small image fields, which are fused to form an overall image, it is possible to perform tissue scanning of large samples. For the experiment, as shown in Fig 1, the sample was translated with a xy-stage. The detection system was mounted over the sample for this purpose. Homogeneous illumination of the sample is necessary to make a quantitative statement about the presence of fluorophore enrichments. This was studied by surveying blind tissue samples.

### In vivo evaluation of the developed laser scanner and comparison with a planar imaging system in a prostate-tumor-bearing animal model

One great advantage of the newly developed optical laser scanner is its mobility compared with existing systems. Therefore, the developed system could be easily transported to the Bruker preclinical imaging reference center site in Mannheim to compare its potential under in vivo conditions with a preclinical fluorescence imaging system, the In-Vivo Xtreme (Bruker, Ettlingen, Germany). In the in vivo experiments, two tumor-specific fluorescently labeled contrast agents bearing different tumor-targeting molecules were used. These molecules were bombesin_7-14_ (BBN_7-14_), which binds to the gastrin-releasing peptide receptor (GRPR), and Lys-urea-Glu (LUG), which addresses the prostate-specific membrane antigen (PSMA). The molecules were assembled on the surface of gold nanoparticles (AuNPs) [46] together with SIDAG dye [absorbance: 600–800 nm/emission: 750–820 nm] to visualize GRPR- and PSMA-positive tumors. The in vivo experiments were performed using seven male SHO mice (Crl:SHO-*Prkdc*^*scid*^*Hr*^*hr*^) obtained from Charles River Laboratories (Sulzfeld, Germany) [47,48]. All experiments were performed according to the local regulations concerning the care and use of laboratory animals (approval number 35–9185.81/G-206/15, Regional Board Karlsruhe, Germany). During the experiments, every effort was made to minimize suffering of the animals. In a pilot study, three healthy mice were examined in both scanner systems. For tumor induction, approximately 5 × 10^6^ cells (GRPR-positive PC3 or PSMA-positive LNCaP cells) in sterile PBS (100 μL) were injected into the left thigh when the mice were 6–8 weeks old. Mouse health and tumor growth were checked daily until the tumors reached a diameter of 4–5 mm (2–3 weeks for PC3, up to 8–12 weeks for LNCaP). GRPR- or PSMA-specific AuNPs were then injected intravenously into the tail vein, and the in vivo distribution was confirmed after 1, 3, 6, 24 and 48 h via optical imaging with both scanner systems. The in vivo detection was not disturbed by background fluorescence. The observed tumor to muscle ratio of 2.5 : 1 was sufficient to reliably differentiate the tumor from surrounding tissue [46]. All injections and measurements of mice were performed under anesthesia (1.5–3% isoflurane/O_2_, 2–3 L/min). For this purpose, the developed optical measurement system was equipped with an additional anesthesia system and a warming pad for in vivo animal optical imaging.

## Results

**Fig 2:**
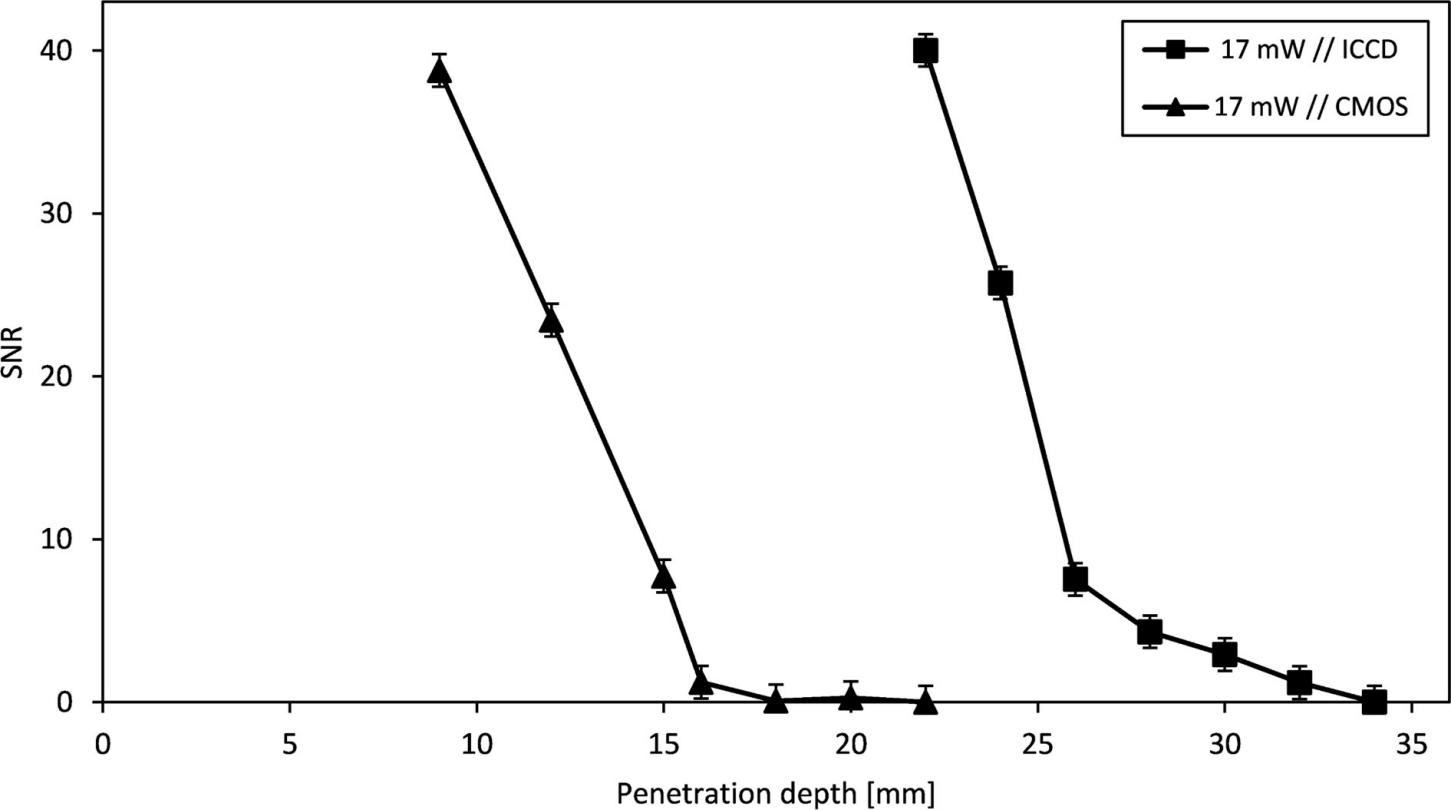
Depiction of obtained signal-to-noise ratios (SNR) imaging tumor phantoms at different depths under the tissue surface.

Experiments to detect fluorescence originating from deep tissue layers were performed to determine the maximum penetration depth in tissue achievable using the developed laser scanner. Fig 2 shows the fluorescence SNR as a function of the penetration depth obtained using the previously described experimental conditions and the developed imaging setup as well as data obtained using the CMOS camera for direct comparison of the imaging systems under the same experimental conditions. The average SNR of the phantoms at different depths under the surface are plotted. The SNR was calculated as the ratio of signal levelto noise level. The detection limit is reported as a signal level that exceeds three times the standard deviation of the noise level. The detection limit of the reference 16-bit camera system was 16 mm. Detection at a tissue depth of up to 30 mm was possible using the intensified CCD (ICCD) camera.

**Fig 3:**
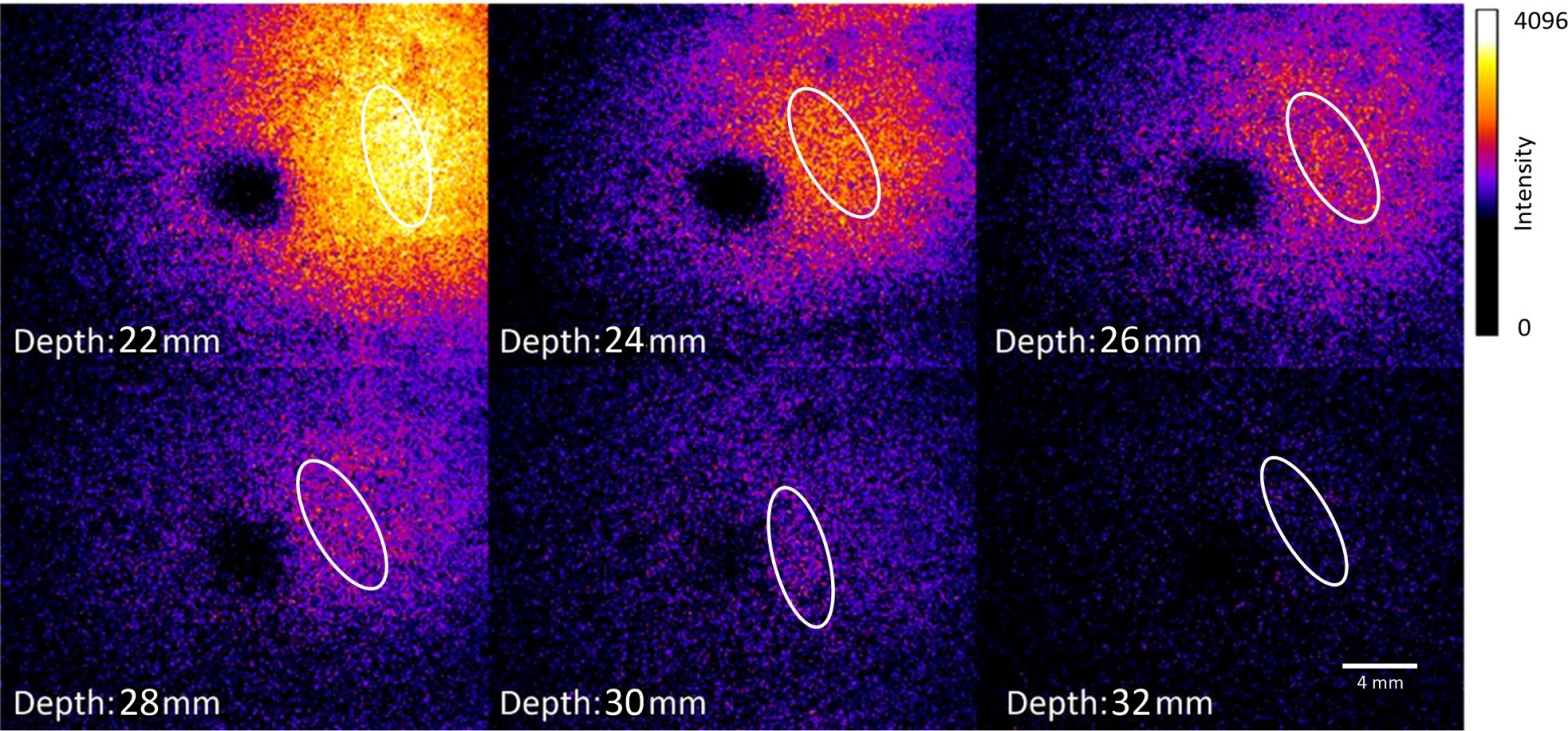
Depiction of the results obtained for ICG-labeled tumor phantoms measured in animal tissue.

Fig 3 shows the results of the second experiment, in which fast fluorescence detection was performed under real-time conditions. For these experiments, the previously described ICG-labeled tumor phantoms were measured in animal tissue at an exposure time of 50 ms with a penetration depth of up to 32 mm. The fluorescence excitation laser was focused on an area adjacent to the measured tumor phantoms. Within the tissue, the excitation light exhibits a spatial distribution due to multiple scattering effects. The scattered excitation light causes fluorescence emission. The fluorescence emission of the labeled tumor phantom is shown in Fig 3. The black area in the center of the image is created by the light trap located immediately in front of the detector, which suppresses the detection of the focused excitation light spot on the sample surface. However, these black spots do not affect the image quality when the sample is scanned several times from different perspectives. Although this requires more time, the device is still much faster than the comparison device (In-Vivo Xtreme). This is clearly demonstrated in the following section, in which a surface scan of a tissue sample was performed. Since every point of the sample is measured several times from different positions, sufficient image information is available at each location. With an increase in the number of individual recordings, the information content can be further increased.

As expected, a decrease in the detected fluorescence was observed with increasing penetration depth. Detection was possible up to a penetration depth of 32 mm. This is a significant increase in the penetration depth compared with that achieved by noncontact real-time laser-induced fluorescence imaging using low-power CW lasers, which only allow a penetration depth of 5–10 mm in real-time measurements [49,50].

**Fig 4:**
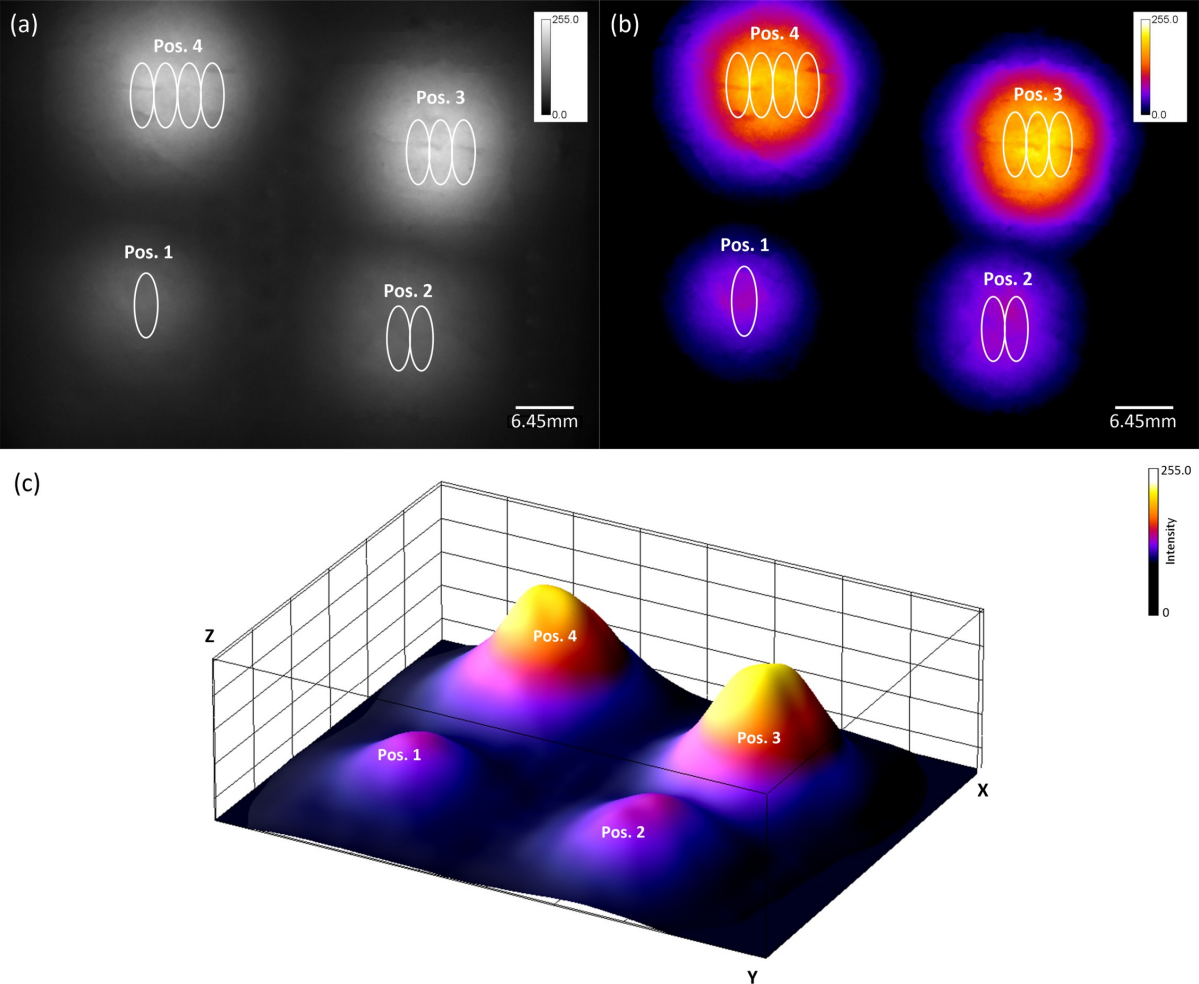
Results of the tissue area scan. (Fig 4A) Tissue scan of an area of 10 cm × 6.6 cm with four differently sized tumor phantoms (phantom sizes: Position 1, 7 × 2 mm; Position 2, 7 × 4 mm; Position 3, 7 × 6 mm; Position 4, 7 × 8 mm) with an exposure time of 100 ms at a depth of 22 mm. (Fig 4B) False-colored image of the scanned tissue sample recorded using the developed laser scanner. (Fig 4C) Three-dimensional surface plot of the detected tumor phantoms.

The following figure (Fig 4A) is a tissue scan of an area of 10 cm × 6.6 cm including four differently sized tumor phantoms (phantom sizes: Position 1, 7 × 2 mm; Position 2, 7 × 4 mm; Position 3, 7 × 6 mm; Position 4, 7 × 8 mm) with an exposure time of 100 ms at a depth of 22 mm. The SNRs in each area are as follows: Position 1, 82; Position 2, 100.5; Position 3. 178.2; and Position 4, 202.2. The tissue scan comprised of a large number of individual images, each with an area of 3 cm × 2 cm, obtained by a lateral offset of each image between the sample surface and the detection area. These images have been linked to a large overlapping image area that can be individually customized to form a fused image. Due to the large overlap and the resulting image information of the overlapping images, which were computed with each other, the black spots resulting from the light trap are not visible in the image. The image stitching and programmatic removal of the light trap spots in Fig 4A were performed with the aid of a software plug-in based on an algorithm by Preibisch et al. [51] using ImageJ v1.51r. The false color image (Fig 4B) illustrates the location of the detected tumor phantoms. The three-dimensional surface plot (Fig 4C) clearly shows the expected fluorescence light emission at the expected points in the image. The collection time was approximately 50 s. The results for the study of the illumination homogeneity (Figs A and B in S2 File) show that homogeneous illumination occurs with a standard deviation of 7.7% relative to the mean illumination average of the sample.

**Fig 5:**
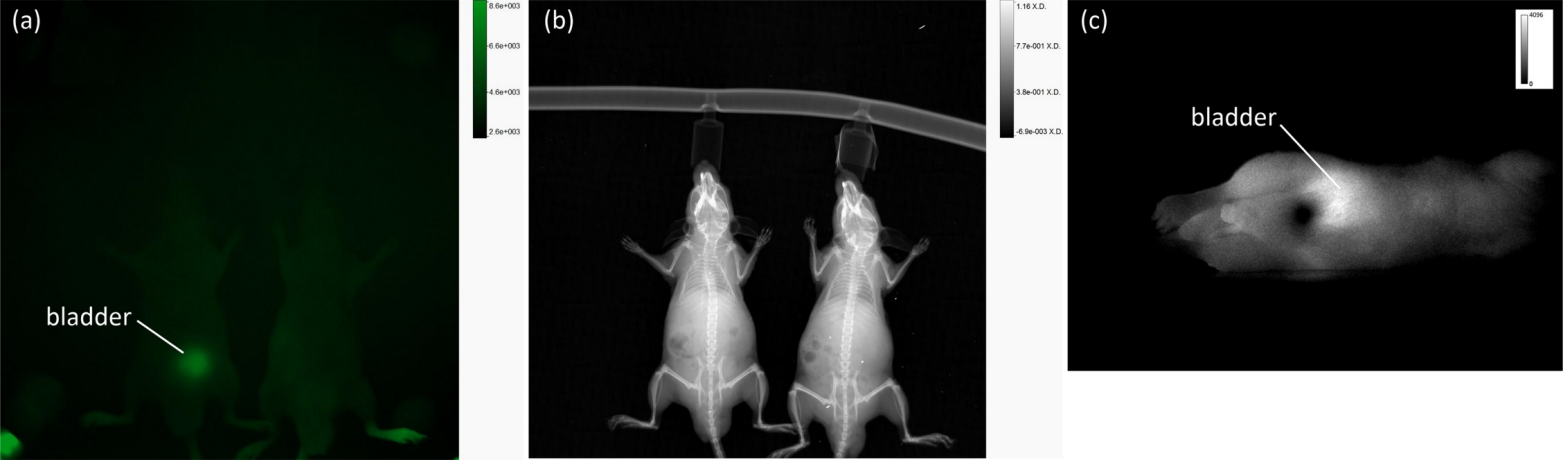
Depiction of the results of the pilot measurements. (Fig 5A) Fluorescence and (Fig 5B) X-ray imaging at 1 h p.i. using AuNP-SIDAG-BBN in healthy mice with the In-Vivo Xtreme system with an exposure time of 60 s. (Fig 5C) Fluorescence imaging at 1 h p.i. using AuNP-SIDAG-BBN in healthy mice with the developed setup and an exposure time of 50 ms.

For the in vivo evaluation and optical setup validation, a comparison was made with a preclinical fluorescence imaging system (In-Vivo Xtreme, Bruker, Ettlingen). First, a pilot measurement was performed in healthy mice using the aforementioned tumor-specific AuNP contrast agents. Rapid renal clearance of the substances was observed, and this is shown in the image in Fig 5A, which was obtained using the In-Vivo Xtreme system, and in Fig 5C, which was obtained with the developed optical setup. Most of the injected fluorescently labeled compounds are located in the bladder (Fig 5A (left mouse)) or were previously secreted Fig 5A (right mouse) at 1 h p.i. Fig 5B shows X-ray images of the same animals also obtained using the In-Vivo Xtreme system. To obtain suitable images using the In-Vivo Xtreme system, an exposure time of 60 s and 2 × 2 binning were necessary. The measurement with the developed laser scanner (Fig 5C) shows the expected results, indicating an accumulation of the fluorophores in the bladder. An exposure time of 50 ms was set without binning. During animal scanning, the structure of the anesthetic system was adjusted. A heat pad was used to stabilize the animals' body temperature during the scans to ensure their welfare.

**Fig 6:**
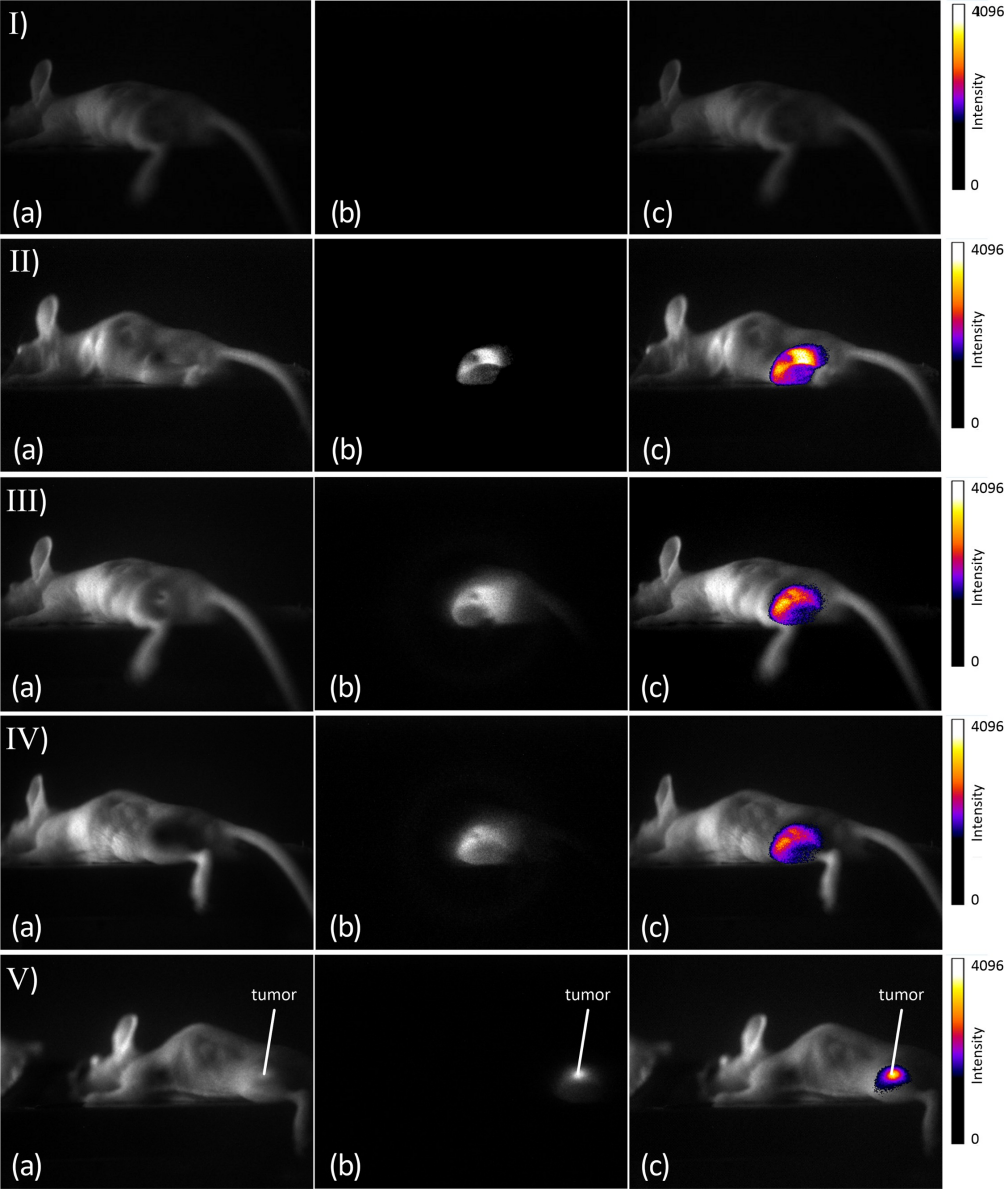
Overlay of Bright Light and Fluorescence Images at the Tumor Site in the Laser Scanner. (Fig 6A) White light, (Fig 6B) fluorescence imaging, (Fig 6C) overlay. I) fluorescence before injection, II) 3 h p.i., III) 6 h p.i., IV) 24 h p.i. and V) 48 h p.i. Measured in the developed laser scanner.

**Fig 7:**
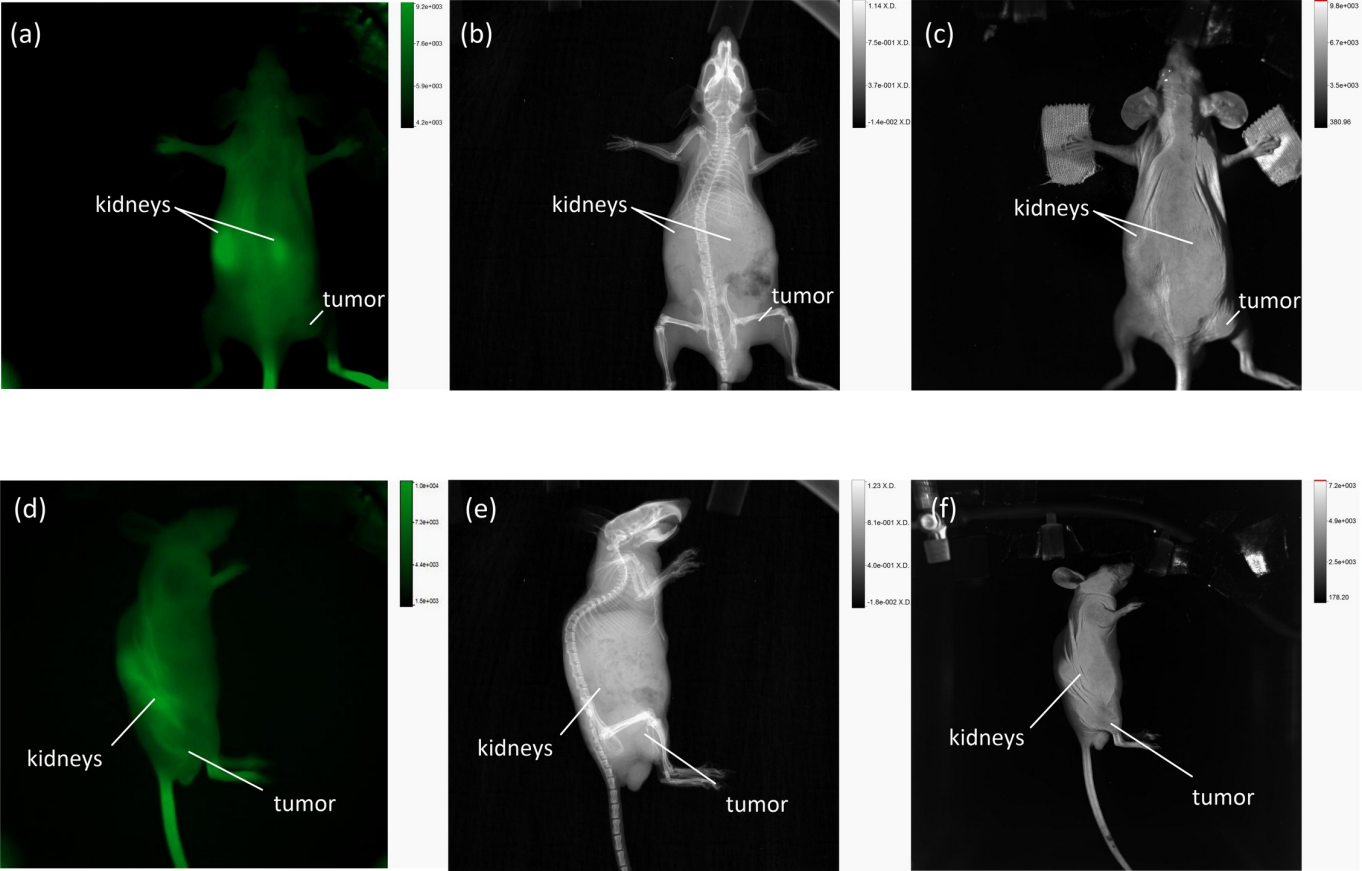
Fluorescence Imaging of AuNP-SIDAG-LUG at 3 h p.i. Using the In Vivo Xtreme System. (Fig 7A) Fluorescence image at an excitation wavelength of 750 nm to 790 nm. (Fig 7B) X-ray imaging. (Fig 7C) Bright field imaging. (Fig 7D) Fluorescence image at an excitation wavelength of 750 nm to 790 nm from the left side of the mouse. (Fig 7E) X-ray Imaging from the left side of the mouse. (Fig 7D) Bright field imaging from the left side of the mouse.

**Fig 8:**
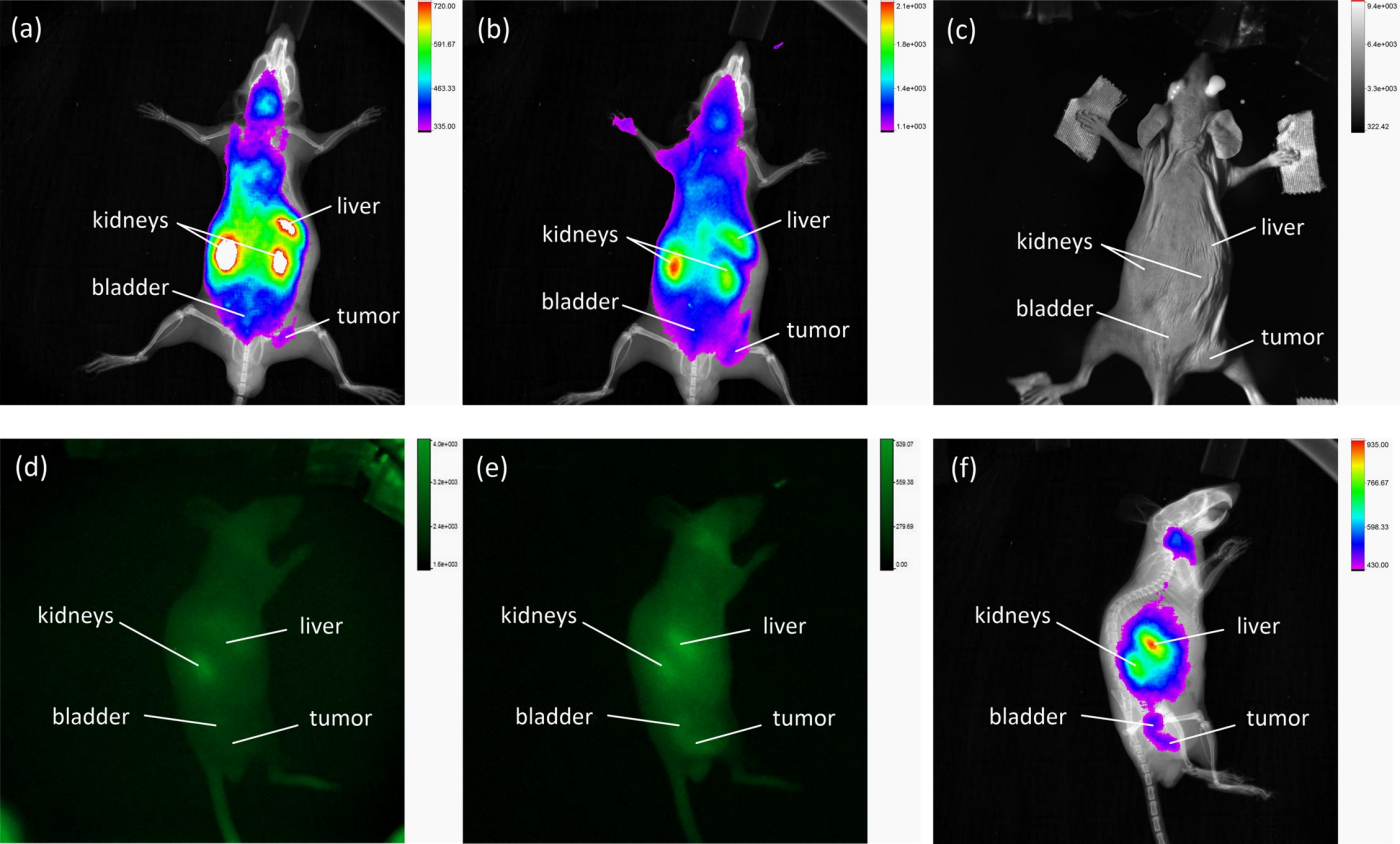
Overlay of X-ray and Fluorescence Imaging at 48 h p.i. Using the In Vivo Xtreme System. (Fig 8A) Fluorescence image of AuNP-SIDAG-LUG obtained at an excitation wavelength of 750 nm to 790 nm and (Fig 8B) 690 nm to 790 nm. (Fig 8C) Corresponding bright field image. (Fig 8D) Fluorescence image obtained at an excitation wavelength of 750 nm to 790 nm from the left side of the mouse. (Fig 8E) Fluorescence image obtained at an excitation wavelength of 690 nm to 790 nm from the left side of the mouse. (Fig 8F) Fluorescence image overlay of AuNP-SIDAG-LUG obtained at an excitation wavelength of 690 nm to 790 nm from the left side of the mouse.

The first evaluations of fluorescent AuNPs in tumor-bearing mice indicated that an accumulation of fluorescence within the tumor occurred at 3 h p.i. at the earliest. Therefore, comparisons of fluorescence imaging of the LNCaP-tumor-bearing mice were performed at 3 h, 6 h, 24 h and 48 h p.i. by measuring the mice first with the In-Vivo Xtreme system and subsequently with the developed laser scanner. The corresponding images were compared on the basis of the signal-to-noise ratios of the fluorescence images. The signal-to-noise ratio of the tumor after 48 h p.i. with the developed scanner was SNR = 56. In contrast, the SNRs were at a fluorescence excitation of 750–790 at SNR = 5.8 and at an excitation of 690–759 at SNR = 9.3.Fig 6A shows a bright-field image, Fig 6B the corresponding fluorescence image and (Fig 6C) an overlay of the bright-field and fluorescence images of AuNP-SIDAG-LUG at the tumor site in the newly developed laser scanner. The field of view was approximately 120 mm x 93 mm. In image II) at 3 h p.i., most of the fluorescence is observed in the bladder. Until that time, no accumulation is found in the tumor; the same observation is shown in Fig 7A–7F, which depicts the corresponding images obtained using the In-Vivo Xtreme system at 750 nm to 790 nm (Fig 7A), X-ray imaging (Fig 7B), bright-field imaging (Fig 7C), fluorescence image at the excitation wavelength of 750 nm to 790 nm from the left side of the mouse (Fig 7D), and X-ray imaging from the left side of the mouse (Fig 7E) and bright-field image from the left side of the mouse (Fig 7F). Fig 6 III) (6 h p.i.) and IV) (24 h p.i.) show a decrease in the fluorescence signal. At 48 h p.i., a significant accumulation of fluorescent particles can be detected at the tumor site (Fig 6V). Fig 8 shows the corresponding images obtained using the In-Vivo Xtreme System at 750 nm to 790 nm (Fig 8A and 8D) and 690 nm to 790 nm (Fig 8B and 8E). The corresponding bright-field image is shown in the right panel (Fig 8C). Fig 8F shows the fluorescence image overlay of AuNP-SIDAG-LUG at an excitation wavelength of 690 nm to 790 nm and an X-ray from the left side of the mouse. The newly developed laser scanner shows the expected significant accumulation of the fluorescent agent in the tumor after 48 h p.i [46].

## Discussion

We have shown the efficient and reliable localization of cyanine- and SIDAG dye- based contrast agents in vitro and in vivo with two imaging systems at different settings. Fig 2 shows the detection limit for the 16-bit camera used as the reference imaging system, which was approximately 16 mm in depth in tissue. In contrast, the detection limit for our modified imaging setup is approximately 32 mm. The developed laser scanner enables a higher SNR than the cooled 16-bit CMOS camera. By combining the image intensifier with a 12-bit CCD camera via a fiber optical connector, the dynamic range of the camera is increased. The image intensifier acts as a preamplifier and increases the dynamics of the detection system. Therefore, extremely weak light signals can be detected. In general, the detection properties are influenced by many different factors. These include the exposure time and the intensity of the excitation light source, which leads to an increase in the intensity of the detected fluorescence upon increase. The concentration of the fluorescent dye, which exhibits the best emission properties at optimum concentration, with these influences being specifically investigated (Figs A-D in S1 File). Additional information about the system properties and the linearity of the developed laser scanner can be found in the supporting informations. Further improvements of the mentioned optical design limits may be achievable. In principle, the developed laser scanner employed in this study could be used to achieve a signal amplification of up to a factor of 1000, depending on the applied voltage of the intensifier. Thus, space-resolved single-photon counting should also be possible. The light trap was developed to achieve this amplification factor because the amplifier should not be overexposed, as this would destroy the amplifier. Therefore, an additional light trap on the detection site is necessary to completely suppress the detection of the excitation light. This is shown by Fig B in S3 File which displays an image with and without a light trap. Another advantage of the light trap is the suppression of the direct reflex, which is consequently not visible in the detection unit. Only light that has passed the tissue is recognized. For physical reasons, reflections, background light and other optical disturbances are amplified in addition to the signal to be detected. To prevent this, the light-disturbing effects were minimized using a massive blocking filter stack in front of the detection system. Unlike various other approaches on fluorescence imaging [18,34], focused excitation illumination is used for this application. Due to the focusing, a high-energy input occurs on a small sample volume. This results from the radiant intensity, which is defined by the quotient of the radiant power and the solid angle. The small diameter of the excitation light spot thus allows a higher irradiance compared to larger spot diameters since the absolute input energy of the system's light source is concentrated on a smaller area. The dependencies of the detectable fluorescence intensity of the introduced radiation power of the excitation light source are shown in the supporting information in Fig B in S2 File. In addition, the light trap should not be too large, since with increasing size of the light trap a loss of the field of view occurs. Therefore, a compromise was necessary between the size of the light trap respectively of the excitation light spot and the field of view. To minimize the extent of the focused beam due to structural differences in the height of the sample surface, a lens with a long focal length (1000 mm) was used for focusing. With a difference of ± 40 mm, the beam expansion is approximately 1 mm. The size of the light trap was chosen so that it can compensate for differences of ± 50 mm. The suppression realized by the localized shuttering of this single spot solved the problems associated with overexposure and the resulting gain limit. This enabled a higher gain voltage to be used for the image intensifier, which consequently increased the signal amplification and SNR. The type of excitation illumination can be influenced by the image representation, and the image information can be almost inverted in extreme cases. The contours, structural bumps and slanted edges are dark or even lighter depending on the angle. In addition, there is an oval excitation spot magnification at a too flat angle of incidence. This in turn affects the size of the light trap. The chosen angle of incidence of the excitation illumination is a compromise between avoiding the direct reflection incident on the detector and the still-existing illumination from the bright field. The illumination from the side at an angle of 35° causes the direct reflections hitting the detector to be reduced. However, there is still a relatively vertical bright illumination of the object. Homogeneous illumination of the sample is necessary to make quantitative statements regarding the presence of tumors. The surface scan (Figs A and B in S2 File) and the subsequent calculation of the individual images show that the sample illumination is homogenous, with a standard deviation of 7.7% relative to the mean illumination of the sample. The homogeneity can be further increased by increasing the number of image recordings made during a scan. This allows a quantitative statement to be made about the presence of tumors. In contrast to the planar imaging system used for comparison, the developed optical setup can only map a small subject field of the image. However, this is not a disadvantage because the laser scanner can also scan larger areas. Due to the short exposure times, which are in the ms range per image, larger areas can be imaged as quickly as with planar imaging systems, which require exposure times in the range of 60–120 s for comparable images. The current system was designed to study smaller samples. It represents a compromise between the size of the sample to be examined and the spatial resolution. With the aid of image stitching, theoretically, the image area can be magnified to an infinite extent. The limitations here are in the mechanic, time and price expenses. For the investigation of larger samples, the field of view could be adjusted, but this would decrease the spatial resolution. The system offers another advantage. In the In Vivo Xtreme system examination (Fig 8B), the tumor is very difficult to detect because the kidneys and liver strongly over-radiate the tumor. This is because the conventional lighting systems are tuned to the homogeneous illumination of the largest possible object fields (19 cm x 13 cm In vivo Xtreme, Bruker). The object to be examined is excited at once for fluorescence emission. To avoid loss of information, the exposure time is set to the area with the highest fluorescence emission, such as liver and kidney, in Fig 8. In this case, areas with low fluorescence emission lose much of their dynamics, which lies in the lower third of the images, by the fluorescence influences from other areas. In contrast to the investigation with the developed imaging system, the tumor can be clearly recognized by the selective excitation shown in Fig 6C. The excitation illumination is specifically tuned to the small subject matter field by focusing. Through to the customized spot lighting, the dynamics can be adapted to any area of interest and cross-influences caused by fluorescence emission from other areas are avoided. This offers the advantage that the fluorescence is excited only in the observed image detail and does not cause overirradiation in other fluorescent image areas in which fluorescence is enriched, such as the kidney or liver. The main advantage of the developed laser scanner is that, using the stitching algorithm, the spatial resolution can be increased several times over that of planar imaging systems, which produce only one image. The image detail on the sample in the individual images can be zoomed in up to the diffraction limit. The working principle can also be adapted to other wavelength ranges. If the setup and the fluorescent dye are adjusted accordingly, the system could also work in other wavelength ranges [50,51,52,53,54]. The laser scanner developed in the study presented here can, as shown, work alone as well as in combination with existing systems, demonstrating its versatility.

## Conclusions

This quantitative fluorescence measurement method, presented in combination with the light trap and filtering strategy, is promising for use in tissue diagnostics using optical fluorescence imaging. The characterization and quantitative analysis of fluorescently labeled tissue in the first optical window was possible through the applied noncontact, real-time laser-induced fluorescence imaging approach. Moreover, in contrast with other clinically established systems, such as CT or MRI, which are routinely used in tumor imaging, the developed laser scanner is simple and inexpensive and can therefore find wide application in tumor diagnostics. Nevertheless, it is not intended to replace but to supplement established imaging modalities, e.g., using these systems for intraoperative imaging. The advantages of the developed detection system are the high SNR and the possibility of increasing the spatial resolution. The use of a small animal rotation system, as used in In Vivo Xtreme, would theoretically also allow the performance of optical tomography. Furthermore, the depth inside the tissue from which the fluorescence can be measured in a contactless manner with the use of this laser scanner should allow preclinical in vivo studies not only in mice but also in the whole rat body using an animal rotation system. From the technical side, a reduction of the blind spot on the detector could occur. For this, the excitation spot size must be further reduced. By this step, the diameter of the light trap could be further reduced. Another way to reduce the blind spot would be a multiple exposure with minimal lateral offset of the optic. The light trap is always a compromise between penetration depth and collection efficiency, because the light trap creates a distance between excitation and detection side. As a result, only light that has penetrate the tissue is detected. Further improvements are conceivable and possible in terms of dye optimization as well as suppression of the scatter caused by surroundings, off-focal-plane light, and secondary emission from the tissue. In this context, further investigations on the various tissue influences (vessel, nerve) within the penetration route should be conducted. These tissues have different densities and thus also influence the scattering and absorption behavior of the fluorescence measurement [7, 8]. Two of the primary objectives during the development of the described laser scanner were to ensure the simplicity of the technology used and its user-friendliness. Overall, this is an easy-to-use, energy-efficient, affordable, and compact system compared to established systems. The proposed technique enables the rapid measurement of fluorescence. Therefore, the laser scanner could also be used to selectively investigate specifically labeled near-surface tumors in humans in real time without causing a dose burden due to ionizing radiation. Other possible fields of application are the detection of near-surface breast cancer, other soft tissue carcinomas, and lymph nodes affected with metastases. Additional applications can be envisioned for the detection of chemical and biological processes involving luminescent or fluorescent substances, e.g., detecting concentration profiles in liquid films or pathogens in wounds and foods. Furthermore, the high measuring speed enables new applications of fluorescence imaging analysis due to the realization of real-time monitoring.

## Supporting information

S1 File Validation of the developed non-contact cw fluorescence imaging laser scanner. (DOCX)

S2 File Examination of sample illumination homogeneity. (DOCX)

S3 File Light path structure of the developed application. (DOCX)
