# Supplementary material for: Noncontact recognition of fluorescently labeled objects in deep tissue via a novel optical light beam arrangement
Source: PLoS One. 2018 Dec 19;13(12):e0208236. doi: 10.1371/journal.pone.0208236 (PMC6300195; doi:10.1371/journal.pone.0208236)
Supplement: S1 File — (DOCX) [file pone.0208236.s001.docx]

## Supporting information

Validation of the developed noncontact CW fluorescence imaging setup with an optimized optical light beam arrangement

The measurements described below were performed to validate the developed setup. For this purpose, the dye concentration, illumination intensity, exposure time and other variables were varied. The variation in fluorescence intensity at a penetration depth of 22 mm is shown as a function of exposure time in Fig S1A. The intensities measured after a 17 mW excitation for various exposure times (5, 10, 20, 50, 80, 100, and 200 ms) were compared with the corresponding dark currents. The relationship between the intensity and exposure time is clearly linear.

The linearity of the fluorescence intensity variation with the excitation intensity in Fig S1B demonstrates that optimal combinations of the laser power and exposure time could be chosen to overcome overheating and motion blur.

Fig S1C depicts the dependence of the fluorescence intensity on the ICG concentration; this was analyzed to determine the ICG detection limit at a laser power of 17 mW and to assess the capabilities of the system at the optimal ICG concentration of 3.125 μg ICG/1 mL H_2_O. At higher ICG concentrations, the signal is reduced by self-quenching. As expected from the known excitation and emission spectra of ICG, the fluorescence increases linearly at low concentrations and then becomes saturated.

Figure S1D shows the relationships of penetration depth and signal-to-noise ratio at various concentrations. The different concentrations in Fig S1D are given in μg ICG/1 mL H_2_O with an exposure time of 150 ms and a laser power of 17 mW.

Supporting information figures





**Fig S1A**





**Fig S1B**





**Fig S1C**

**

**

**Fig S1D**
