# Supplementary material for: Noncontact recognition of fluorescently labeled objects in deep tissue via a novel optical light beam arrangement
Source: PLoS One. 2018 Dec 19;13(12):e0208236. doi: 10.1371/journal.pone.0208236 (PMC6300195; doi:10.1371/journal.pone.0208236)
Supplement: S2 File — (DOCX) [file pone.0208236.s002.docx]

## Supporting information

Examination of the sample illumination homogeneity

Examination of the sample illumination homogeneity (Fig S2A and B) shows that homogeneous illumination occurs with a standard deviation of 7.7% relative to the mean illumination average of the sample. The tissue scan comprises 121 individual images. Each image has an image area of 3 cm × 2 cm. These images were stitched with an image area overlap of 2/3 to form an overall image. The image stitching of Fig S2 was performed using ImageJ v1.51r.


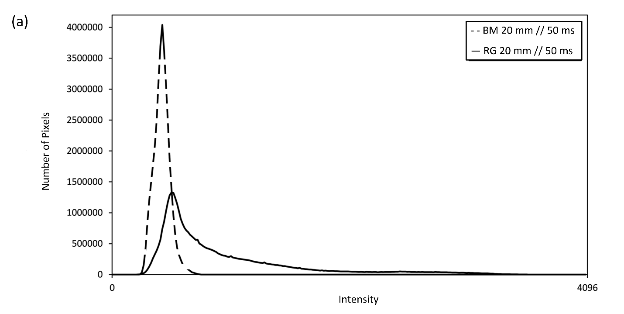


**Fig S2A**

**
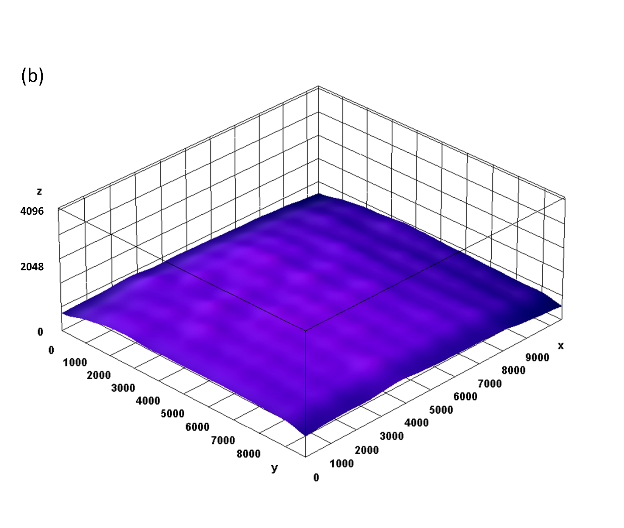
**

**Fig S2B**
