# Supplementary material for: Noncontact recognition of fluorescently labeled objects in deep tissue via a novel optical light beam arrangement
Source: PLoS One. 2018 Dec 19;13(12):e0208236. doi: 10.1371/journal.pone.0208236 (PMC6300195; doi:10.1371/journal.pone.0208236)
Supplement: S3 File — (DOCX) [file pone.0208236.s003.docx]

## Supporting information

Light path structure of the developed application

Fig S3A provides an overview of the light path structure of the developed imaging system. The light trap is an intervention to reduce the detection of excitation light. This is located directly in front of the sensor, centrally. This prevents the light spot incident on the object from overexposing the image and thus falsifying the measurement. In addition, a permanent overexposure of the detector at full gain level would destroy it**.** Fig S3B on the left shows an image in which the excitation radiation is not covered by the light trap. In the right figure, however, the excitation is covered by the light trap.


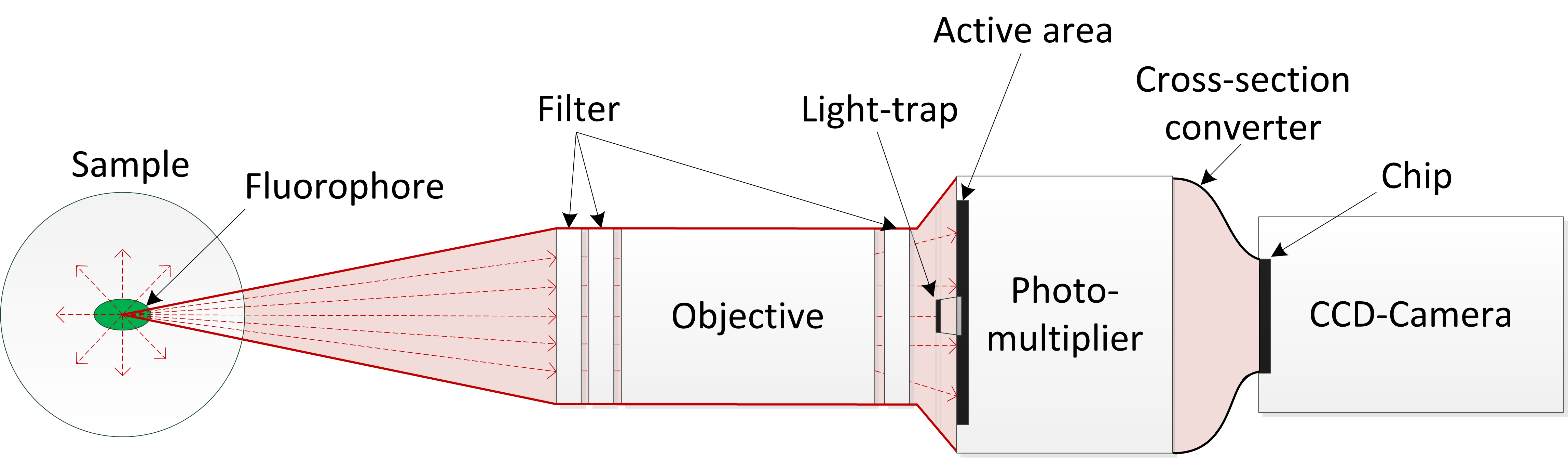


**Fig S3A**


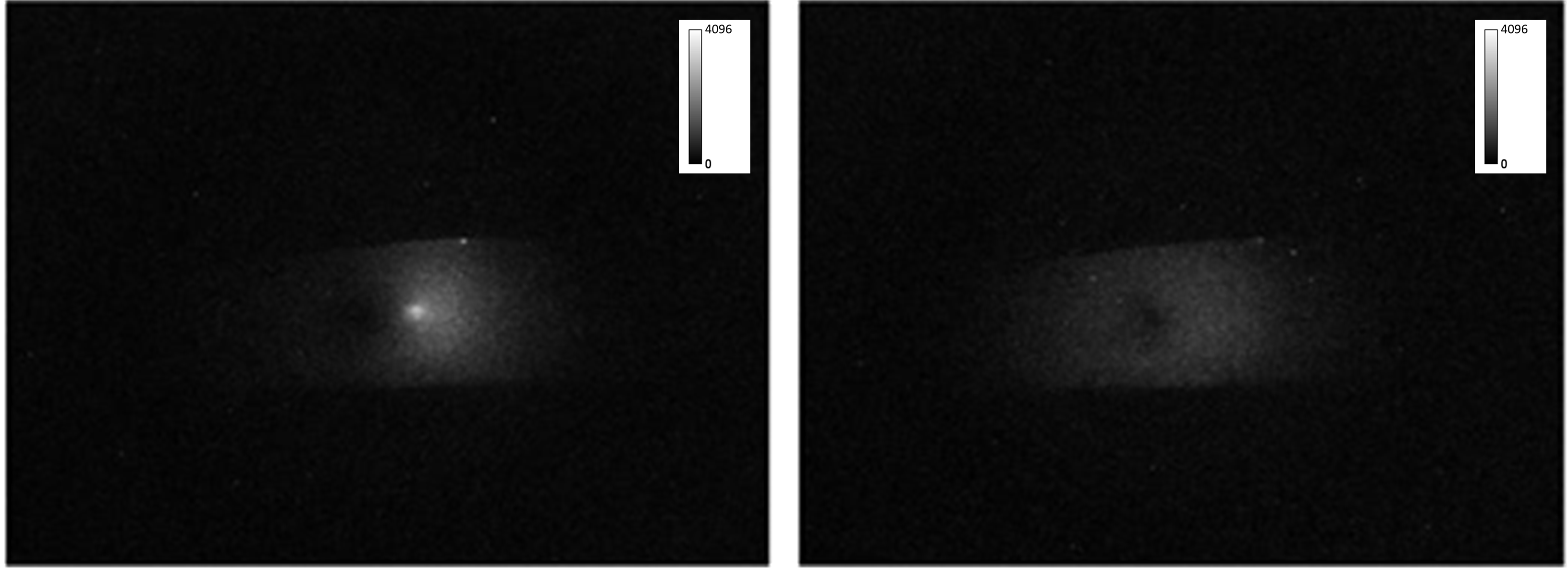


Fig S3B
